# Comparative analysis of trends and determinants of anaemia between adult and teenage pregnant women in two rural districts of Ghana

**DOI:** 10.1186/s12889-019-7603-6

**Published:** 2019-10-26

**Authors:** Millicenta K. M. Ampiah, Jerry J. Kovey, Charles Apprey, Reginald A. Annan

**Affiliations:** Department of Biochemistry and Biotechnology, College of Science, Kwame Nkrumah University of Science and Technology, Kumasi, Ghana

**Keywords:** Pregnancy, Anaemia, Iron deficiency, Supplementation

## Abstract

**Background:**

The prevalence of iron deficiency anaemia remains high in pregnant women and the situation may be worse for pregnant adolescents. This study aimed to comparatively analyse the trends and determinants of anaemia between adult and teenage pregnant women in rural Ghana.

**Methods:**

A retrospective study design was employed. Data including primarily pregnancy history, haemoglobin levels and anaemia status were collected from the manual medical records of 1002 women stored in the repositories of two rural district hospitals in the Ashanti Region of Ghana over the years 2011–2015. Data was analysed using chi-square analysis, t-test, two-way ANOVA and binary logistic regression. Bivariate and multivariate analyses were also done to establish associations and predictors of anaemia.

**Results:**

An overall drop in mean haemoglobin from 11.1 g/dl in 2011 to 10.6 g/dl in 2015 was observed for adults, while an overall increase from 9.4 g/dl to 10.2 g/dl occurred in teenagers. Further, anaemia prevalence declined at the 36th week of pregnancy, and from 2011 to 2015, for both teenagers and adults. Among factors tested for association with haemoglobin levels using bivariate and multivariate analyses, gestational age alone was significant (*P* = 0.028). Between-subject effects determined using 2-way ANOVA indicated year of pregnancy alone, as well as in combination with age group (F = 3.1, *P* = 0.019) significantly affected haemoglobin levels. From binary regression analysis, BMI (OR 0.967, 95% CI:0.936–0.999, *P* = 0.042), gestational age (OR 1.058, 95% CI:1.013–1.106, *P* = 0.011) and pregnancy year (OR-2012(0.402); 2013(0.53); 2014(0.569); 2015(0.817), 95% CI: 2012(0.256–0.631); 2013(0.338–0.829); 2014(0.366–0.886); 2015(0.501–1.333), *P* = 0.001) were found to be significant predictors of anaemia at first antenatal visit. Also, the trimester of reporting for antenatal care, specifically the second trimester (OR-0.261, 95% CI: 0.072–0.951, *P* = 0.042) and the pregnancy year (OR-2012(0.235); 2013(0.206); 2014(0.530); 2015(0.222), 95% CI: 2012(0.101–0.545); 2013(0.081–0.522); 2014(0.197–1.428); 2015(0.049–1.018), *P* = 0.003) were more significant at the 36th pregnancy week.

**Conclusions:**

Although the trends observed were decreasing in both adults and teenagers in the years reported, anaemia levels remained high for each year in either group. Anaemia, therefore remains a major health problem, especially in the areas studied, and antenatal interventions need a second look to know what might make them more effective.

## Background

Anaemia is a condition which results from an inadequate number of erythrocytes and consequently a resultant decreased ability to carry oxygen to meet the physiological demands of the body [3]. It is characterized by a decrease in the amount of haemoglobin (Hb) present in an individual [20]. It is estimated that anaemia affects 29% (496 million) of non-pregnant women, 38% (32.4 million) of pregnant women aged 15–49 years and 43% of children worldwide [25, 30]. According to the World Health Organization (WHO), an anaemic condition may be termed severe, mild or moderate, depending on the Hb status of the individual. The Hb levels of these forms of anaemia in pregnant women are ≤10.9 g/dl (mild anaemia), ≤9.9 g/dl (moderate anaemia) and < 7 g/dl (severe anaemia) (WHO, 2004). The causes of anaemia vary: they may be- genetic, such as haemoglobinopathies and thalassemia; infectious, such as malaria, intestinal helminths and chronic infection or nutritional, notably nutritional deficiencies in iron, folate, vitamins A and B12 and copper (WHO, 2004). According to the WHO report in 2008, about four hundred different kinds of anaemia have been recorded but the most common forms of anaemia include: iron deficiency anaemia, anaemia due to folic acid or vitamin B12 deficiency, haemolytic anaemia, and sickle cell anaemia.

Iron deficiency anaemia is known to be the most common form of anaemia. Statistics indicate that globally, more individuals live with iron deficiency anaemia than any other medical condition [2]. In Ghana, work done by Stevens et al.*,* [25], showed that the prevalence of iron deficiency anaemia in pregnant women far exceeds 50%. Iron deficiency anaemia in pregnancy is a pressing issue and must be completely done away with, as it affects pregnancy outcomes, resulting in serious infant and maternal complications such as maternal and perinatal mortality, low birth weights as well as postpartum haemorrhage [16, 24]. Some studies have shown that anaemia during pregnancy alone accounts for about 23% of the indirect causes of maternal deaths in developing countries [8]. Preterm and low birth weight are still the leading causes of neonatal deaths in developing countries, contributing to 30% of the deaths [19]. Anaemia has also been associated with increased risk of intrauterine fetal deaths (IUFD), as well as, intrauterine growth restriction (IUGR) which poses a risk for stunting among children of less than two years [15, 21].

Iron and folic acid supplementation (60 mg of elemental iron and 400 μg folic acid for all pregnant women) was put in place by the WHO in 2001 to curtail anaemia in pregnant women [28]. Despite the overwhelming consequences, not much work has been done on the prevalence, trends and associated factors of iron deficiency in pregnant women in Ghana, especially after the implementation of the iron-folate supplementation. One of the few reports existing after the implementation, that is the Ashanti Health Report in 2010, indicated that despite these interventions, anaemia took its place as the second leading contributing cause of death in the Ashanti Region.

Moreover, a study by Glover-Amenyo et al.*,* [17] reported that rural dwelling was a strong predisposing factor for anaemia in pregnancy, as against urban dwelling, further suggesting a need to focus on rural areas as a focal point for studying the anaemia incidence and management. However, most rural regions and districts in Ghana have limited or lack empirical data on anaemia and its contributing factors to serve as a justification for interventions. Routine medical data are collected on pregnant women in Ghana, but such data have not been analysed in a way that allows better understanding of what might be factors that contribute to anaemia in pregnant women and how anaemia may be ameliorated. Also, studies [7, 23] have reported a higher incidence of anaemia in pregnant teenage girls against their adult counterparts, primarily owing to the higher iron demand during adolescence as a result of growth increase, as well as a rather poor nutrient intake in this age group. These imply the need to stratify analysis according to age groups for adequate analysis and intervention which might be an important step in directly combating maternal mortality, exacerbated by adolescent pregnancy, as defined by the 2015 UN sustainable development goals.

This study thus sought to analyse the trends and associated factors of anaemia between adult and teenage pregnant women in two rural districts, Ahafo Ano South and Asante Akim South, in the Ashanti region of Ghana. These districts were interesting for such studies as the dietary patterns of the population, generally consisting of a high consumption of starchy foods- such as plantain, yam, maize, were representative of that of most rural parts of Ghana. Moreover, with these characteristics they served as the ideal sites for studying iron deficiency anaemia, which is largely linked to nutritional intake.

## Methods

### Study/ recruitment sites

Seven (7) health centres were selected from each of the districts, Ahafo Ano South and Asante Akim South for the retrieval of data. For Asante Akim South district, the selected health centers were Banka Health Centre, Juaso District Hospital, Bompata Health Centre, Komeso Health Centre, Obogu Health Centre, St Roses Health Centre and Ofoase Health Centre. For Ahafo Ano South district, the selected health centres were Mankraso Government Hospital, Pokukrom Health Centre, Mpasaso Health Centre, Sabronum Health Centre, Wioso Health Centre, St. Edward’s Clinic and St. John’s Clinic. Each health centre served as a direct point of referral for antenatal care in each sub-district within the two districts.

### Description of study population

The Ashanti region is the third largest region in Ghana after the Northern and the Brong Ahafo regions by land size. Asante Akim South District, with its capital, Juaso, is one of the administrative districts of Ashanti, with a population of about 150,000 people (according to the 2000 population census) (MOFA, 2018). The district is strategically located at the entry and exit point to the Ashanti Region and is mainly rural and agricultural in nature with over 60% of the farmers being tenant farmers. Ahafo Ano South district, also in the Ashanti region, has a population of 133,632 representing about 3.7% of the region’s total population of 3,612,950 (MOFA, 2018). The district is particularly noted for a high growth rate due to high fertility rate, as well as its agricultural and mining activities.

### Study design

The study was a retrospective study conducted between October, 2016 and May, 2017.

### Sampling and data collection

The sampling frame covered the entire available antenatal attendance records of pregnant women who visited the selected health centres (above described) between 2011 and 2015. The sample consisted of those with complete records in the ANC units and record keeping sections of the health centres at the time of the study. Missing or incomplete records were excluded from the study.

Data collection was done in two forms. Firstly, all complied data were collected from the yearly ANC summaries of the electronic medical records of the health centres in each of the districts for the review years, 2011 to 2015. The data retrieved included total anaemia cases per year and total Hb level checked per year.

In the second phase of data collection, cluster random sampling was used. Extrapolating from the available number of complete records, individual data of a total of 1000 pregnant women (both teenagers and adults), specifically 501 per district were randomly collected from ANC records of the antenatal care units of the main district health centers, i.e. Juaso District Hospital (Asante Akim South) and Mankraso Government Hospital (Ahafo Ano South), for each of the review years. Randomly collected data was later segmented according to age during analysis. The main district centres were the focus in the second phase of data collection due to the inability to obtain the manual records of the sub-district health centres. This involved the use of a comprehensive data collection form designed to capture the Hb levels of subjects, their ages, their blood pressures, their HIV statuses, sickling statuses, weight, height, parity, gestational period, and other special conditions observed. Data was retrieved for years, 2011 to 2015.

#### Data analysis

Microsoft Excel was used in the random sampling of the patient registration numbers during the second level of data collection. GraphPad Prism vs 8, was used in chi-square analysis, t-test analysis and calculation of *p*-values. Comparison of mean Hb between teenagers and adults and within each group at different years and stages of pregnancy were conducted using t-test, assuming normal distribution. Normal distribution was tested using normality and lognormality tests (Shapiro- Wilk test) with GraphPad Prism vs 8. Using SPSS vs, 20, bivariate and multivariate analyses, two-way ANOVA and binary logistic regression were carried out to determine the factors associated with Hb levels in the participants, effects of multiple factors on Hb levels and the predictors of anaemia at antenatal registration and at 36th week of pregnancy respectively. Anaemia was classified based on Hb levels. Hb levels below 11 g/dl were classified as anaemic (WHO, 2004).

## Results

### Comparison of adult and teenage participants by haemoglobin, anthropometrics, blood pressure and other characteristics

From the chi-square analysis at 95% confidence interval (Table 1), the mean age for the teenagers was 17.57 years and that of the adults was 28.5 years. The adults had significantly higher gravida (*p* < 0.001), mean Hb at registration (*p* = 0.028), body weight (p < 0.001) and BMI (p < 0.001) than the teenagers. Mean Hb at the 36th week of pregnancy, systolic blood pressure and gestational age between the teenage and adult pregnant women were however not significantly different.

**Table 1:** Comparison of mean demographics, Hb and pregnancy history between teenage and adult groups

		N	Mean	Std. Deviation		
					F	Sig.
Age (years)	Teenager	119	17.57	1.37	381.07	< 0.001
	Adult	879	28.53	6.10		
	Total	998	27.22	6.75		
Height (cm)	Teenager	112	156.30	15.23	2.71	0.1
	Adult	842	158.06	9.88		
	Total	954	157.85	10.65		
Gravida	Teenager	101	0.37	0.75	105.57	< 0.001
	Adult	843	2.26	1.84		
	Total	944	2.06	1.85		
Gestational Age (weeks)	Teenager	121	17.29	7.88	0.08	0.784
	Adult	879	17.05	9.12		
	Total	1000	17.08	8.97		
Reporting Trimester	Teenager	122	1.85	0.64	0.02	0.885
	Adult	880	1.84	0.76		
	Total	1002	1.84	0.74		
Hb at registration (g/dl)	Teenager	121	9.79	1.97	4.85	0.028
	Adult	874	10.73	4.62		
	Total	995	10.61	4.40		
Hb at 36th week (g/dl)	Teenager	25	10.53	2.50	0.02	0.893
	Adult	213	10.58	1.65		
	Total	238	10.58	1.75		
Weight	Teenager	122	55.30	8.78	34.33	< 0.001
	Adult	874	61.32	10.86		
	Total	996	60.59	10.81		
Systolic Blood Pressure (mmHg)	Teenager	118	104.51	16.44	1.05	0.306
	Adult	829	106.01	14.70		
	Total	947	105.82	14.92		
BMI (kg/m2)	Teenager	112	22.43	3.60	21.22	< 0.001
	Adult	836	24.45	4.45		
	Total	948	24.21	4.40		

The mean ages of the teenage and the adult groups were 17.57 and 28.53 years respectively. Based on chi-square analysis of individual data of a total of approximately 1000 individuals, significant differences were observed with respect to gravida, Hb levels at registration, weight and BMI between teenage and adult groups. The ratio of teenagers to adults per 1000 records collected was about 1:9. Hb- Haemoglobin; BMI- Body mass index

### Five-year trend of mean haemoglobin at registration, week 36 of pregnancy and mean BMI between teenage and adults

**Fig. 1:**
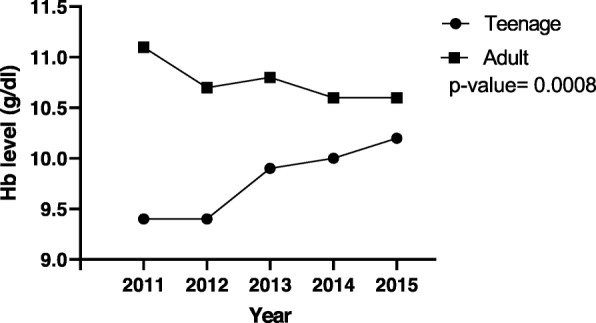
Five- year trend of mean Hb at antenatal registration for teenage and adult pregnant women. A decline from 11.1 to 10.6 is seen from 2011 to 2015 for the adult group while an increase from 9.4 to 10.2 is seen for the teenage group. Hb- haemoglobin; blue- teenage; red- adult. *P* value< 0.05 (significant)

**Fig. 2:**
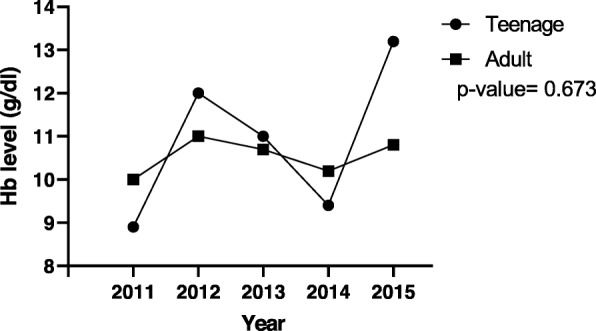
Five- year trend of mean 36th week Hb for teenage and adult pregnant women. The mean Hb of the adult group hovered around 10 g/dl -11 g/dl whle for teenagers, a maximum of 13.2 g/dl was recorded in 2015, after a series of rise and decline from 8.9 g/dl in 2011. Hb- haemoglobin; blue- teenagers; red- adults. P value> 0.05 (not significant)

**Fig. 3:**
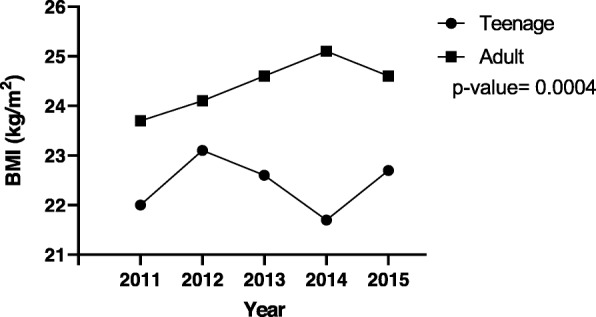
Five- year trend of mean BMI for teenage and adult pregnant women. The mean BMI for the teenage group was lower than that of the adult group across the years (21.7–23.1 vs 23.7–25.1 kg/m^2)^. However, in both cases, BMI generally fell within the normal range (18.5–24.9 kg/m^2^). BMI- body mass index; blue- teenage; red- adult. P value< 0.05 (significant)

Figures 1, 2 and 3 illustrate respectively, 5-year mean Hb at registration, 5-year mean Hb at 36th week and 5-year mean BMI trends between the pregnant teenagers and adults. The results (Fig. 1) showed a fluctuation over the 5 years but an overall drop of mean Hb from 11.1 g/dl in 2011 to 10.6 g/dl in 2015 for the adult population while for the teenagers, there an overall increase from 9.4 g/dl to 10.2 g/dl in the same period. For the results of the five-year trend of mean Hb level at the 36th week of pregnancy (Fig. 2), the trend for the adult pregnant women showed very little variation (stayed around 10 g/dl) in comparison to the trend for teenagers, over the same period. Specifically, for teenagers, the mean Hb level peaked at 2015 (13.5 g/dl) and 2012 (12 g/dl) and had its least value in 2011 (8.9 g/dl). For BMI, most values fell within the normal range, that is, 18.5 to 24.9 kg/m^2^ (Fig. 3). The trend for the adult population increased steadily from 23.7 kg/m^2^ in 2011 to a peak of 25.1 kg/m^2^ in 2014, which subsequently dropped to 24.6 kg/m^2^ in 2015. The trend for teenagers showed more fluctuation, peaking at 23.1 kg/m^2^ in 2012 and reaching a minimum of 21.7 kg/m^2^ in 2014.

### Five-year trend of anaemia per age group, at registration and at week 36 of pregnancy

**Fig. 4:**
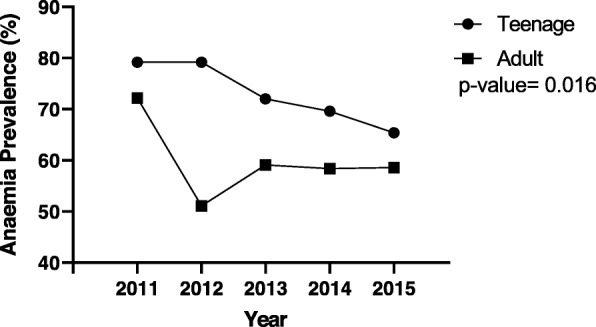
Five-year trend of anaemia prevalence for teenage and adult groups. For both teenage and adult groups, anaemia prevalence decreased from 2011 to 2015 (79.20–65.40 vs 72.20–58.60%). However, in the adult group, a slight increase was seen from 2012 to 2013 but this was followed by a gradual decline till 2015. blue- teenage; red- adult. P value< 0.05 (significant)

For the five-year anaemia trend for both pregnant teenagers and adults presented in Fig. 4, anaemia prevalence was generally higher in the teenage group than the adults throughout the five-year period, although anaemia prevalence dropped from about 80% to about 70% in the teenagers and from 70 to 60% in the adult group. Additionally, in the adult group, the least anaemia prevalence was recorded in 2012 (50%).

### Analysis of mean haemoglobin at antenatal registration and at the 36th week of pregnancy

Table 2 shows the comparison of mean Hb at antenatal registration and at the 36th week of gestation for the combined group (teenage+ adult) and for each group (teenage or adult). Using t-test analysis at 95% confidence interval, results indicated no significant difference in the mean Hb levels at ANC registration and by 36 weeks of pregnancy in each case (that is teenagers only, adults only and the combined group). Further analysis in the teenage and adult groups showed a significant difference in the mean Hb levels of the two groups at ANC registration (*p* = 0.028); but not at 36 weeks (*p* = 0.893). The correlation coefficients obtained showed a weak but positive correlation between Hb at antenatal registration and at 36 weeks for the teenage group (R = 0.423, *p* = 0.039), adult group (R = 0.179, *p* = 0.001) and both groups (R = 0.2, p = 0.001).

**Table 2:** Hb levels per age group at ANC registration and at the 36th week of gestation

Groups		Mean	N	Std. Deviation	Mean difference within group	P value	Within group correlation coefficient	P value
Combined group (Teenage+ Adult)	Hb at registration	10.20	231	1.8	0.26	0.070	0.22	0.001
	Hb at 36th week	10.50	231	1.7				
Teenage	Hb at registration*	10.03	24	2.0	0.46	0.376	0.42	0.039
	Hb at 36th week^	10.50	24	2.5				
Adult	Hb at registration*	10.30	207	1.8	0.23	0.123	0.18	0.001
	Hb at 36th week^	10.55	207	1.6				

*p-value- analysis of the mean difference between teenage only and adult only groups for Hb levels at antenatal registration = 0.028

^*p*-value- analysis of the mean difference between teenage only and adult only groups for Hb levels at the 36th week of gestation = 0.893

No mean difference was found between teenagers and adults at ANC registration and at the 36th week of gestation, when combined and analysed separately. A significant difference was found between the Hb levels of teenagers and adults at ANC registration. Further analysis of the correlation between Hb levels at ANC registration and the 36th week of gestation showed a weak but positive correlation in both teenagers and adults, when analysed together and separately. Hb- Haemoglobin; ANC- Antenatal care

### Analysis of factors associated with haemoglobin levels

Further, bivariate and multivariate analyses were conducted to analyse the effect of various variables (factors) on Hb. The results of a multivariate analysis, conducted at 95% confidence interval (Table 3) showed that amongst various factors that were tested (BMI, District, Gestational age, age group, year, sickling status, age group and year, age group and sickling status, year and sickling status, age group and year and sickling status), gestational age alone was significantly associated with the Hb levels.

**Table 3:** Bivariate and multivariate analyses of the factors associated with Hb levels in the participants

Effect		Value	F	Hypothesis df	Error df	Sig.
Factor	Pillai’s Trace	0.001	.112b	1	197	0.738
Factor * BMI	Pillai’s Trace	0.001	.111b	1	197	0.74
Factor * District	Pillai’s Trace	0.019	3.715b	1	197	0.055
Factor * Gestational age	Pillai’s Trace	0.024	4.910b	1	197	0.028
Factor * Age group	Pillai’s Trace	0.002	.319b	1	197	0.573
Factor * Year	Pillai’s Trace	0.013	.670b	4	197	0.613
Factor * Sickling status	Pillai’s Trace	0	.025b	1	197	0.874
Factor * Age group	Pillai’s Trace	0.033	1.695b	4	197	0.153
Factor * Age group * Sickling status	Pillai’s Trace	0	.b	0	0	.
Factor * Year * Sickling status	Pillai’s Trace	0.012	.580b	4	197	0.678
Factor * Age group * Year * Sickling status	Pillai’s Trace	0	.b	0	0	.

The association between multiple factors (BMI, district, gestational age, age group, year, sickling status, age group and year, age group and sickling status, year and sickling status, age group and year and sickling status) and the Hb levels of subjects (factor) was tested by bivariate and multivariate models. Gestational age alone was found to be significantly associated with the Hb levels of subjects (pregnant women of all age groups). BMI- body mass index; Hb- haemoglobin

The results of the tests of between-subject effects, conducted in 2-way ANOVA (95% confidence interval), displayed in Table 4, indicated that the year of pregnancy and age group together had a significant effect on Hb levels of subjects. In addition to this, the year of pregnancy alone also had a significant effect on Hb levels (*p*-value = 0.001).

**Table 4:** Tests of between-subject effect of multiple factors on Hb levels

Tests of Between-Subjects Effects					
Measure: Hb					
Transformed Variable: Average					
Source	Type III Sum of Squares	df	Mean Square	F	Sig.
Intercept	589.003	1	589.003	178.552	< 0.001
BMI	5.24	1	5.24	1.588	0.209
District	5.459	1	5.459	1.655	0.200
Gestational Age	0.373	1	0.373	0.113	0.737
Age group	0.947	1	0.947	0.287	0.593
Year	62.294	4	15.573	4.721	0.001
Sickling	3.871	1	3.871	1.173	0.280
Age group * Year	39.659	4	9.915	3.006	0.019
Age group * Sickling	0	0	–	–	–
Year * Sickling	19.566	4	4.892	1.483	0.209
Age group * Year * Sickling	0	0	–	–	–
Error	649.857	197	3.299		

Amongst a variety of factors tested -BMI, district, gestational age, age group, year, sickling status, age group and year, age group and sickling status, year and sickling status, age group and year and sickling status- the year of pregnancy alone and in combination with age group, were significantly in terms of determining Hb levels of subjects. Hb- Haemoglobin; BMI- Body mass index

The results of binary logistic regression analyses to determine the predictors of anaemia at antenatal registration (Table 5) and at week 36 of gestation (Table 6) showed the significant predictors of anaemia at antenatal registration to be BMI (*p*-value = 0.042), gestational age (p-value = 0.011) and pregnancy year. Specifically, as compared to others, 2011, 2012 and 2015 were associated with lowered odds of anaemia. On the other hand, the significant predictors of anaemia at 36th week of gestation, were the trimester of first reporting to the hospital, with the second trimester being the most significant, as well as the year of pregnancy, such that using 2011 as reference, later years were less associated with anaemia. Anaemia was defined using Hb levels of less than 11 g/dl. Hb levels of 11 g/dl and above were defined as non-anaemic (WHO, 2004).

**Table 5:** Binary regression to determine the predictors of anaemia at antenatal registration

	B	P value	Exp (B)	95% Confidence Interval for Exp(B)	
				Lower	Upper
BMI	−0.034	0.042	0.967	0.936	0.999
Gestational Age	0.057	0.011	1.058	1.013	1.106
Year		0.001			
2012	−0.911	0	0.402	0.256	0.631
2013	−0.635	0.005	0.53	0.338	0.829
2014	−0.564	0.013	0.569	0.366	0.886
2015	−0.202	0.419	0.817	0.501	1.333

Significant predictors of anaemia at antenatal registration were BMI, gestational age and pregnancy year. Hb < 11 g/dl = anaemic; Hb levels ≥11 g/dl = non-anaemic (WHO, 2004). Hb- haemoglobin; BMI- Body mass index. B = slope. Exp (B)- odds ratio

**Table 6:** Binary regression showing predictors of anaemia at the 36th week of gestation

VaVariable	B	P value	Exp (B)	95% Confidence Interval for Exp (B)	
				Lower	Upper
Trimester (1)		.125			
Trimester (2)	−1.342	.042	.261	.072	.951
Trimester (3)	−2.330	.063	.097	.008	1.137
Year		.003			
2012	−1.448	.001	.235	.101	.545
2013	−1.579	.001	.206	.081	.522
2014	−.634	.210	.530	.197	1.428
2015	−1.504	.053	.222	.049	1.018

At the 36th week of gestation, significant predictors were ANC reporting in the second trimester and the year of pregnancy. Hb < 11 g/dl = anaemic. Hb levels ≥11 g/dl = non-anaemic (WHO, 2004). Hb- Haemoglobin; BMI- Body mass index. B = slope. Exp (B)- odds ratio

## Discussion

The aim of the study was to analyse the trends and associated factors of anaemia between adult and teenage pregnant women in two rural districts, Ahafo Ano South and Asante Akim South, in the Ashanti region of Ghana. A study of the demographics indicated that the average age difference between teenagers and adults was about 10 years. This further showed that the teenagers had carried fewer pregnancies than the adults (lower gravidae index). They also had lower mean body weight, BMI and mean Hb at first antenatal services. These implied that the teenagers were more likely at risk of a poorer health status. Previous reports also indicated an increase in anaemia occurrence and severity with a higher gravida index [4]. In this study, although the adults had higher gravida, the teenagers were more anaemic. This may be due to poorer nutrition in the younger age group, and an increased demand for nutrients in the teenage group as compared to their adult counterparts [7] during pregnancy, since teenagers are more likely to use their nutrients for their own growth and development, before the foetus.

As part of antenatal services offered to these pregnant women, Hb levels are assessed on their first attendance to antenatal care and at 36 weeks of gestation. A comparison of the mean Hb levels between each year within each age group and between the two over the five-year period reviewed showed that between 2011 and 2015, mean Hb at registration reduced in adults by 0.3 g/dl while the teenagers increased by 0.8 g/dl. At 36 weeks of pregnancy however, the trend for adult pregnant women showed very little variation (stayed around 10 g/dl). Also, the Hb levels of the teenagers showed similarity to that of the adults, at 36 weeks of pregnancy, thus suggesting an improvement in the Hb levels of the teenage group. The reasons for this are not obvious, but it is possible that antenatal interventions were more effective in teenage group. Future studies especially, randomised controlled trials, are required to better understand the reasons behind earlier mentioned observations.

In general, the range of values obtained for anaemia prevalence over the years (that is 50–80%) was comparatively higher than that recorded in previous studies in other parts of Africa (42.7%) (Tunkyi and Moodley, 2015), as well as WHO estimates for anaemia prevalence (56%) in low and middle-income countries [13]. These imply that although measures have been put in place to curtail anaemia, there is a high chance that expected results are not being achieved. The likely unresponsiveness to iron-folate supplementation in pregnant women has in previous studies been attributed to the insufficient intake of supplements, ongoing blood losses due to parasitic infections, concomitant folate or B_12_ deficiency, or a rare autosomal disorder known as IRIDA [11, 22]. These results suggest the need for another look at anaemia intervention methods, to achieve the expected outcome, in adults and teenagers alike. A systematic study focused on the effectiveness of the supplementation programme might also be required to confirm and justify the possible need for restructuring the current anaemia supplementation programme.

Generally, mean Hb levels of both teenagers and adults were found to be higher by 36th week of pregnancy than at antenatal registration. Also, positive correlations were observed between Hb at antenatal registration and at 36th week gestation, indicating that pre/ early pregnancy Hb levels, largely determined anaemia occurrence during latter stages of pregnancy. Further, the above observation also implied that the effectiveness of iron-folate supplementation and other interventions to advance blood Hb levels during pregnancy was largely dependent on pre-pregnancy Hb levels. Thus, implying the need for a focus on pre-pregnancy measures to prevent anaemia in pregnancy. Measures such as encouraging food fortification and diversification which have been shown to reduce anaemia in women of child-bearing age, should be encouraged [6]. A follow up study looking at the individual dietary intake of women during pregnancy may be necessary to ascertain and inform the enforcement of these measures.

As mentioned earlier, the Hb levels of the pregnant women were higher at the 36th week, suggesting an improvement over the course of pregnancy. However, according to findings, the average age of antenatal registration was 17 weeks of gestation, implying that pregnancy cases were presented to the health centres within the second trimester. Effective supplementation covers the course of the pregnancy (folic acid in first trimester, iron+ folic acid in second and third trimesters) and thus, although an improvement was observed, late reporting of the pregnant women to the health centers may have reduced the overall efficiency of the supplementation [1]. This prompts the need to promote early visits to antenatal services by pregnant women in order to realize the full benefit of antenatal interventions, and ultimately reduce maternal mortality and other undesirable birth outcomes [9, 18].

In our study sample, BMI fell within the normal range (i.e 18.5 kg/m^2^ to 24.9 kg/m^2^) and showed no associations with anaemia in late pregnancy. However, an association between BMI and anaemia occurrence during antenatal registration was observed. Ugwuja et al., [27] observed no association between BMI and anaemia in pregnant women but our findings suggest that the weight status of the women matters, especially at the earlier stage of pregnancy. The difference may be due to the stratification of the occurrence of anaemia based on the stage of pregnancy (early versus late stage) and therefore more studies employing such stratification methods might be necessary to get a clear picture of the association between the two.

Analyses of the factors associated with anaemia indicated that both the year of pregnancy and the age group of participants were co-effective for determining Hb levels, which might be predicted by socio-economic factors such as economic conditions and food availability [7, 14]. This study was performed in two districts and therefore a wider coverage area in future studies might be more adequate to assess the socio-economic determinants of anaemia in rural areas to influence intervention strategies. Such studies and interventions should also consider the type and quality of diet of pregnant women, especially at various stages of the pregnancy to induce the required change in Hb status and thus anaemia levels in pregnant women [5, 12].

Also, from binary regression analysis, significant predictors of anaemia at antenatal registration were different from the predictors at the 36th week of gestation. BMI, gestational age at first antenatal service and year of pregnancy predicted Hb levels at antenatal registration. At the 36th week of gestation, only the year of pregnancy and the trimester of receiving antenatal services, specifically the second trimester, were significant predictors of Hb levels. Such information might be useful in developing a model for studying and monitoring anaemia prevalence and trends.

The devastating effect of anaemia on pregnancy outcomes has been widely recognized globally, and thus is a major setback in eradicating maternal mortality, with reference to the UN 2015 sustainable development goals. Although some studies have reported that anaemia may protect against stillbirth and preeclampsia in pregnancy [10], a large degree of evidence shows its negative effect on maternal and infant health, during and after pregnancy, especially contributing to low birth weight [24]. We suggest long term longitudinal studies on the effect of anaemia on health outcomes to put in place preventive measures against such.

## Conclusion

In conclusion, anaemia prevalence was high at both antenatal registration and the 36th week of gestation. Also, pregnant teenagers and adults were likely to visit antenatal services and start supplementation as late as 17 weeks into pregnancy further reducing the effectiveness of the supplementation programme. The teenage group was found as more anaemic, thus calling for interventions to reduce teenage pregnancies. Policies surrounding education, advocacy and mobilization should be put in place to engage teenagers especially on the medical complications associated with teenage pregnancy. The predictors of low Hb suggest general improvement over the years have led to improved Hb status in the population. Teenagers weight status or BMI should be considered since that also predicts their Hb at antenatal registration.

## Supplementary information

**Additional file 1.** A compilation of the raw data collected from all health centres included in this study. (XLSX 14 kb)

**Additional file 2.** A compilation of the raw data collected from the ANC records of the participants included in this study. (XLSX 78 kb)

## Data Availability

All data generated or analysed during this study are included in this published article [and its supplementary information files [Additional file 1: COMPILED DATA (HEALTH CENTRES)_amended; Additional file 2: RAW DATA_ALL PARTICIPANTS_amended].

## Abbreviations

ANC: Antenatal care
ANOVA: Analysis of Variance
BMI: Body Mass Index
GHS: Ghana Health Service
Hb: Haemoglobin
HIV: Human Immunodeficiency Virus
IRIDA: Iron Refractory Iron-deficiency Anaemia
IUFD: Intrauterine Fetal Death
IUGR: Intrauterine Growth Restriction
MOFA: Ministry of Food and Agriculture
SPSS: Statistical Package for Social Sciences
WHO: World Health Organization
